# Chinese patient with neurofibromatosis-Noonan syndrome caused by novel heterozygous NF1 exons 1–58 deletion: a case report

**DOI:** 10.1186/s12887-020-02102-z

**Published:** 2020-05-01

**Authors:** Zhen Zhang, Xin Chen, Rui Zhou, Huaixiang Yin, Jiali Xu

**Affiliations:** Department of Pediatrics, The First Affiliated Hospital of Bengbu Medical College, Bengbu, 233004 Anhui China

**Keywords:** Neurofibromatosis type 1, Noonan syndrome, Deletion, Heterozygous, Case report

## Abstract

**Background:**

Neurofibromatosis-Noonan syndrome (NFNS) is a rare autosomal dominant hereditary disease. We present a case of NFNS due to the heterozygous deletion of exons 1–58 of the *NF1* gene on chromosome 17 in a 15-month-old boy.

**Case presentation:**

A 15-month-old boy was admitted for motor and language developmental delay, numerous café-au-lait spots, hypertelorism, left blepharoptosis, pectus excavatum, cryptorchidism, secondary atrial septal defect, and UBOs (undefined bright objects) revealed by cranial MRI T2FLAIR in basal ganglia and cerebellum. Using whole exome sequencing, we identified a de novo heterozygous deletion including exons 1–58 of the *NF1* gene.

**Conclusion:**

Although genetic tests are useful tools for diagnosis of NFNS, NF1, or NS, comprehensive analysis of genetic factors and phenotypes is indispensable in the clinical practice. To the best of our knowledge, this case presents the first Chinese NFNS case due to NF1 defects, and the NF1 exons 1–58 deletion-related phenotype is unlike any other reported case.

## Background

Neurofibromatosis-Noonan syndrome (NFNS) is a rare autosomal dominant hereditary disease with clinical characteristics of neurofibromatosis type I (NF1) and Noonan syndrome (NS) [1]. In recent years, there had been reports of NFNS cases related to NF1 gene mutations in foreign countries [2–5], but no cases had been reported in China. We present a 15-month-old boy with NFNS associated with exons 1–58 heterozygous deletion of the NF1 gene on chromosome 17. This case is expected to improve clinicians’ understanding of the disease.

## Case presentation

A 15-month-old boy was admitted to the pediatric clinic of the First Affiliated Hospital of Bengbu Medical College on April 23, 2018, because he could not walk. The medical history reported by the parents was that café-au-lait spots could be seen on the skin of the body and limbs when the child was born. With aging, the spots gradually increased and became larger, and the child’s development was retarded. At admission, the child could not speak, crawl, or walk. The patient was first born child with normal full-term delivery. The birth weight was 3.0 kg. The parents of the child were normal, and there was no family history.

**Fig. 1:**
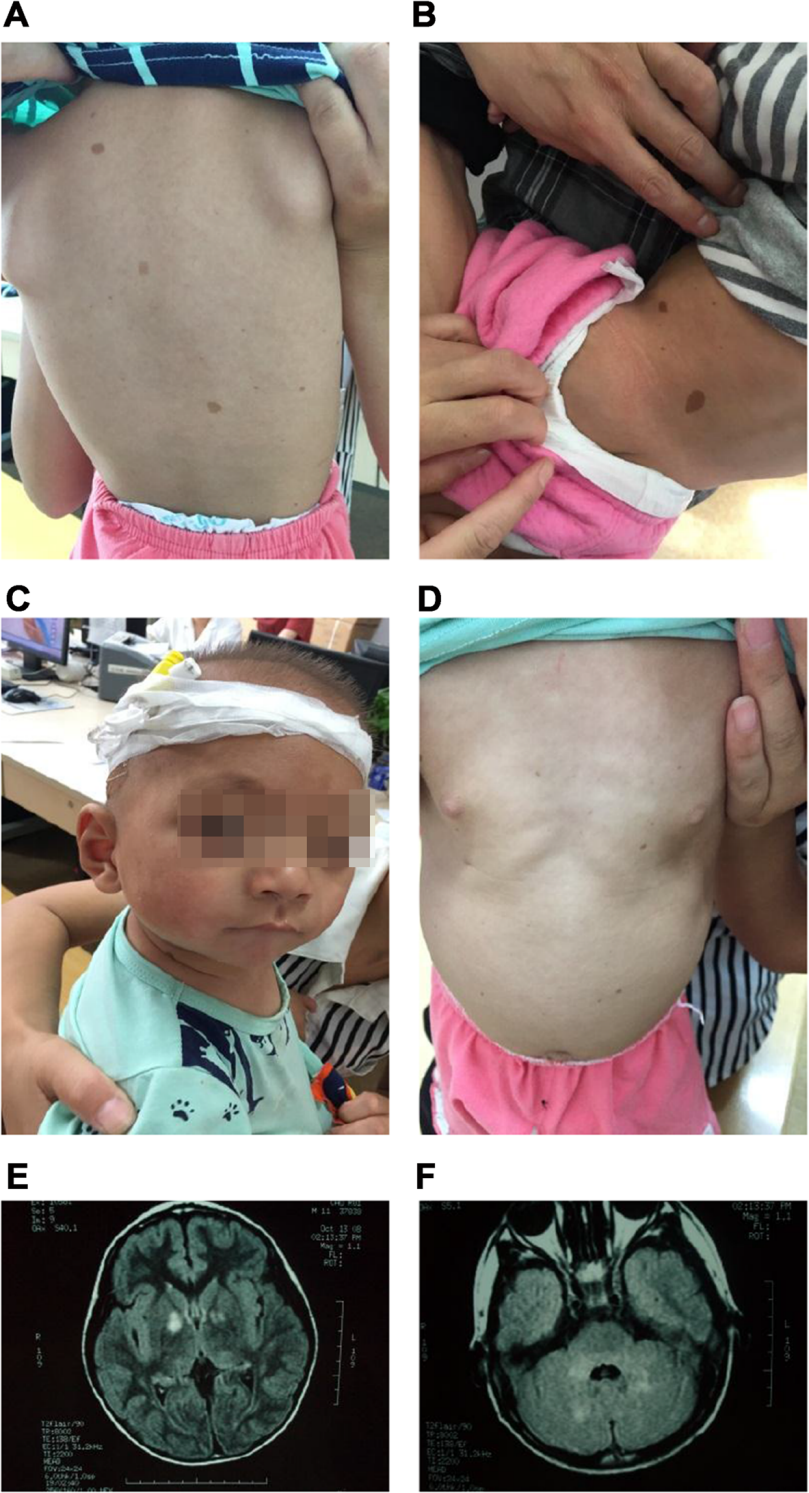
**a**-**b** Café-au-lait spots on the skin. **c** Unusual face, with widely-spaced eyes and left blepharoptosis. **d** Pectus excavatum. **e**-**f** Cranial MRI showing lesions with high signal intensity on T2FLAIR in basal ganglia and cerebellum

Physical examinations showed that body temperature was 36.5 °C, breathing was 24 times/min, pulse was 120 bpm, body mass was 9.2 kg (−1SD to -2SD), body length was 72.5 cm (−2SD to -3SD), and with a conscious mind. Oval café-au-lait spots of different sizes were scattered on the skin of the body and limbs, with light brown and clear boundary, not protruding from the surface of the skin, with a diameter of about 0.5–1.5 cm (more than 6 had a maximum diameter of more than 5 mm) (Fig. 1a-b). He had hypertelorism (widened eyes distance) with left blepharoptosis (Fig. 1c) and congenital pectus excavatum (Fig. 1d). We found the patient grade 2–3 systolic ejection murmurs on the left second intercostal, below the margin of sternum and bilateral cryptorchidism, without hepatosplenomegaly.

The results of tandem mass spectrometry, urinary organic acid analysis, and blood gas analysis, gonadotropin, sex hormones, and plasma testosterone level were normal, and no significant anomaly of eye sight and was found. Result of ultrasonocardiography suggested the patient had secondary atrial septal defect. Cranial magnetic resonance imaging (MRI) showed lesions with intermediate signal intensity on T1WI and high signal intensity on T2WI and T2FLAIR, as known as UBOs (undefined bright objects), and the lesion boundary was not clear (Fig. 1e-f).

**Fig. 2:**
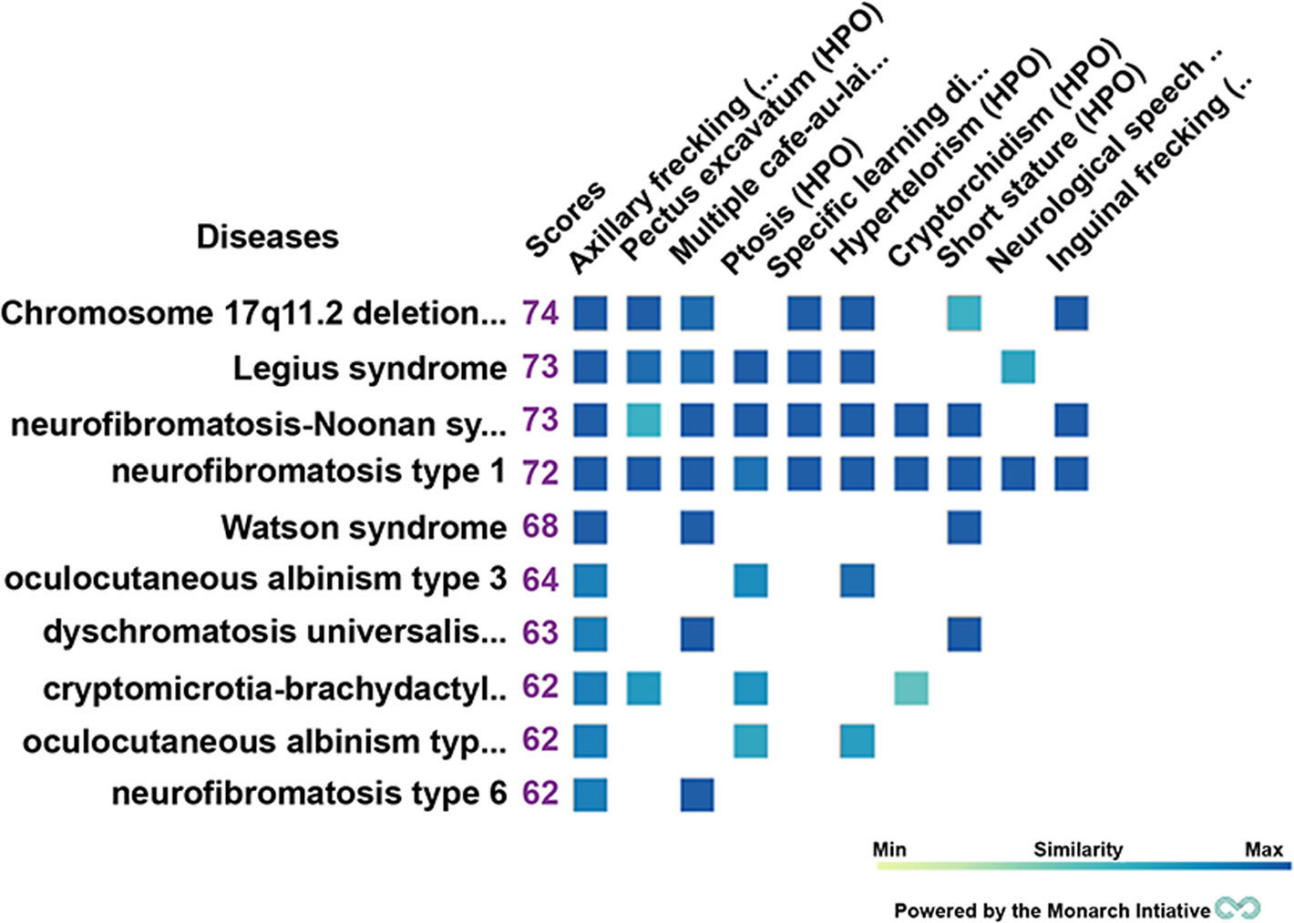
Top 10 diseases screened using online tool, Phenotype Profile Search, The Monarch Initiative. The analysis can be performed on page https://monarchinitiative.org/analyze/phenotypes

To diagnose, we collated the signs and symptoms of the patient into forms of HPO (the Human Phenotype Ontology) and retrieved the top 10 diseases associated with the phenotypes using the online analysis tool, Phenotype Profile Search, provided by The Monarch Initiative (https://monarchinitiative.org/), and it turned out that chromosome 17q11.2 deletion syndrome, Legius syndrome, NFNS, and NF1 are the most likely primary disorders. (Fig. 2).

**Fig. 3:**
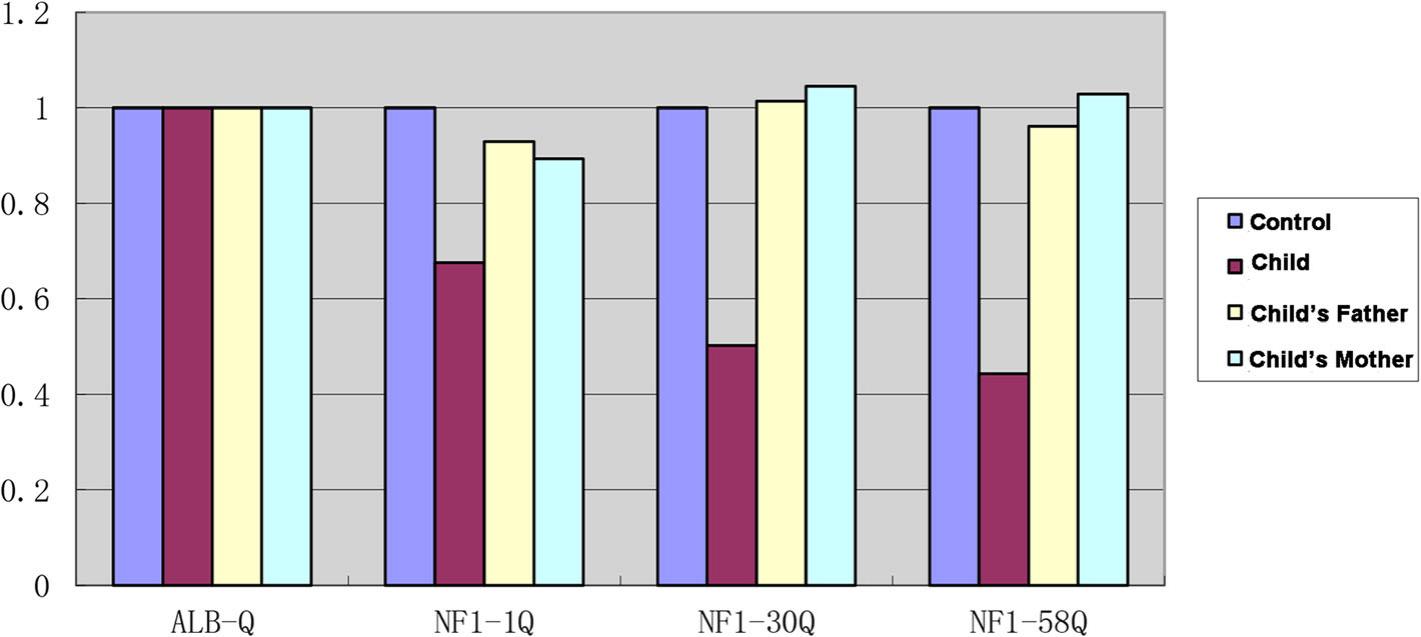
Detection of exons 1–58 of the NF I gene in the proband and his parents. The results showed that the ratio of the copy number of exons 1–58 of the NF I gene in the proband to the normal control was about 0.5, indicating that there was a heterozygous deletion of exons 1–58 of the NF I gene. The ratio of the copy number of exons 1–58 of the NF I gene in the proband’s parents to the normal control was about 1, indicating that the copy number of exons 1–58 of the NF I gene in the proband’s parents was normal

To confirm the findings and differentially diagnose, we performed whole exome sequencing (Deyi Dongfang Translational Medical Research Center, Beijing, China) to find the genetic factors. The targeted sequencing followed the instruction recommended by Illumina using the short reading method to screen single nucleotide variants (SNVs) and short indels (< 50 bp), followed by a comprehensive analysis of variant pathogenicity assessment according to the ACMG clinical practice guidelines, genotype-phenotype matching, and inheritance type confirmed by trio data and Sanger sequencing or real-time PCR (Polymerase Chain Reaction). Based on the sequencing data, however, we found a de novo heterozygous large deletion, including the exons 1–58, in the *NF1* gene, which is not previously documented and it was confirmed by using real-time PCR (Fig. 3). We also analyzed variations in the genes correlated to the RAS/MAPK pathway signaling, *PTPN11*, *SOS1*, *RAF1*, *BRAF*, *SHOC2*, *KRAS*, *HRAS*, *MAP 2 K2*, *PPP1CB*, *RRAS*, and *MAP 2 K1*, and Legius syndrome related *SPRED1*, and no pathogenic or likely pathogenic mutations were found.

At present, the child is undergoing rehabilitation training. Motor development is still retarded. The child shows no other abnormalities.

## Discussion

NFNS is a clinically independent autosomal dominant hereditary disease [6] and it is considered as a RASopathy, which are defined as a group of medical genetic syndromes caused by germline mutations in genes that encode components or regulators of the Ras/mitogen-activated protein kinase (MAPK) signaling pathway, including Noonan syndrome and Noonan-related syndromes [7]. All the phenotypes in our patient can be explained by the RASopathies, e.g., NF1 and NS (Fig. 2), and age and environmental factors may play roles in onset of NFNS phenotype, which makes it difficult to diagnose without genetic testing [7–9].

The result, see Fig. 2, shows that the phenotypes in patients with RASopathies overlap with each other and it lacks distinct genotype-phenotype relationships, however, *NF1* exon 1–58 deletion in our patient may help to understand the genotype-phenotype relationship in NF1-related disorders. Kehrer-Sawatzki H et al. [10] discussed the genotype-phenotype relationships in patients with large *NF1* deletions, as known as Neurofibromatosis type 1 microdeletion syndrome, and the authors suggested that overgrowth or tall-for-age stature, large hands and feet, hyperflexibility of joints, muscular hypotonia, increased numbers of subcutaneous, and plexiform and spinal neurofibromas are most common findings in the patients, however, none of these was found in our patient. On the other hand, some patients with canonical NFNS, characterized by multiple cafe-au-lait macules, absence of neurofibromas, short stature, learning disabilities, pulmonary valve stenosis and features of Noonan syndrome, were found due to specific *NF1* mutations, such as c.2970-2972delAAT deletion or missense variants at codon 1809 [11, 12]. Based on the documented research, the findings, *NF1* exons 1–58 deletion-related phenotype in NFNS patients could be different from that of the other mutations, in our case suggest a new NFNS-related genotype of *NF1* defects.

Unequal meiotic crossover has been reported to be responsible for microdeletions in the *NF1* gene [13],which could explain the mechanism of the deletion in our patient, but the clinical outcomes may vary. Comparing to MRI findings, intermediate signal intensity on T1WI, and high signal intensity on T2WI and FLAIR, and no enhancement, no abnormal signal of space-occupying effect in enhanced scan in NF1 patients [14], results of MRI in our patient showed UBOs (undefined bright objects, nonspecific white matter hyperintensity signals on T2-weighted or fluid attenuated inversion recovery (FLAIR) MRI), which means non-specific pathological changes and etiology. Sabol et al. [15] suggested that UBOs should be considered a criterion for NF1 diagnosis, because UBOs were found in 97–100% NF1 patients, and, in previously reported case [16] and in our patient, UBOs were found in NFNS patients, which provides further evidence that NFNS is a mixed type syndrome of both NF1 and NS.

In addition, NFNS should be differentiated from the Leopard Syndrome. The main characteristics of the Leopard Syndrome are numerous pigmented nevus (similar to “leopard skin” spots), facial features, and cardiovascular and nervous system abnormalities. The Leopard Syndrome is an autosomal dominant genetic disease, mainly caused by *PTPN11*, instead of *NF1*, mutations [17]. Regarding diagnosis of other RASopathies patients with multiple lentigines, genetic tests were needed to exclude candidate RASopathy genes, such as *PTPN11* and *RAF1*, mutations [18].

## Conclusion

This is, as far as we know, the first documented Chinese NFNS case [19], which may indicate the underestimated relevance of NFNS in Chinese patients with NS or NS-like disorders, as known as RASopathies. Genetic test is powerful tool for differential diagnosis in RASopathies patients, however, meticulous identification of symptoms and signs and a comprehensive analysis are also critical in clinical practice. Based on the findings in our patient with novel *NF1* exons 1–58 deletion, it suggests a new genotype-phenotype relationship remained to be clarified by further research.

## Data Availability

The datasets used and/or analyzed during the current study are available from the corresponding author on reasonable request.

## Abbreviations

FLAIR: Fluid attenuated inversion recovery
HPO: The Human Phenotype Ontology
MAPK: Mitogen-activated protein kinase
MRI: Magnetic resonance imaging
NF1: Neurofibromatosis type 1
NFNS: Neurofibromatosis-Noonan syndrome
NS: Noonan syndrome
PCR: Polymerase chain reaction
SD: Standard deviation
SNV: Single nucleotide variant
UBOs: Undefined bright objects.

## References

[1] Opitz JM, Weaver DD (1985). The neurofibromatosis-Noonan syndrome. Am J Med Genet.

[2] Thiel C, Wilken M, Zenker M, Sticht H, Fahsold R, Gusek-Schneider GC (2009). Independent NF1 and PTPN11 mutations in a family with neurofibromatosis-Noonan syndrome. Am J Med Genet A.

[3] Nystrom AM, Ekvall S, Allanson J, Edeby C, Elinder M, Holmstrom G (2009). Noonan syndrome and neurofibromatosis type I in a family with a novel mutation in NF1. Clin Genet.

[4] Yimenicioglu S, Yakut A, Karaer K, Zenker M, Ekici A, Carman KB (2012). A new nonsense mutation in the NF1 gene with neurofibromatosis-Noonan syndrome phenotype. Childs Nerv Syst.

[5] Ekvall S, Sjors K, Jonzon A, Vihinen M, Anneren G, Bondeson ML (2014). Novel association of neurofibromatosis type 1-causing mutations in families with neurofibromatosis-Noonan syndrome. Am J Med Genet A.

[6] Allanson JE, Hall JG, Van Allen MI (1985). Noonan phenotype associated with neurofibromatosis. Am J Med Genet.

[7] Yapijakis C, Pachis N, Voumvourakis C (2017). Neurofibromatosis-Noonan syndrome: a possible paradigm of the combination of genetic and epigenetic factors. Adv Exp Med Biol.

[8] Shilyansky C, Karlsgodt KH, Cummings DM, Sidiropoulou K, Hardt M, James AS (2010). Neurofibromin regulates corticostriatal inhibitory networks during working memory performance. Proc Natl Acad Sci U S A.

[9] Noonan JA, Kappelgaard AM (2015). The efficacy and safety of growth hormone therapy in children with Noonan syndrome: a review of the evidence. Horm Res Paediatr.

[10] Kehrer-Sawatzki H, Mautner VF, Cooper DN (2017). Emerging genotype-phenotype relationships in patients with large NF1 deletions. Hum Genet.

[11] Koczkowska M, Callens T, Gomes A, Sharp A, Chen Y, Hicks AD (2019). Expanding the clinical phenotype of individuals with a 3-bp in-frame deletion of the NF1 gene (c.2970_2972del): an update of genotype-phenotype correlation. Genet Med.

[12] Rojnueangnit K, Xie J, Gomes A, Sharp A, Callens T, Chen Y (2015). High incidence of Noonan syndrome features including short stature and pulmonic stenosis in patients carrying NF1 missense mutations affecting p.Arg1809: genotype-phenotype correlation. Hum Mutat.

[13] Lopez Correa C, Brems H, Lazaro C, Marynen P, Legius E (2000). Unequal meiotic crossover: a frequent cause of NF1 microdeletions. Am J Hum Genet.

[14] Billiet T, Madler B, D'Arco F, Peeters R, Deprez S, Plasschaert E (2014). Characterizing the microstructural basis of “unidentified bright objects” in neurofibromatosis type 1: a combined in vivo multicomponent T2 relaxation and multi-shell diffusion MRI analysis. Neuroimage Clin.

[15] Sabol Z, Resic B, Gjergja Juraski R, Sabol F, Kovac Sizgoric M, Orsolic K (2011). Clinical sensitivity and specificity of multiple T2-hyperintensities on brain magnetic resonance imaging in diagnosis of neurofibromatosis type 1 in children: diagnostic accuracy study. Croat Med J.

[16] De Luca A, Bottillo I, Sarkozy A, Carta C, Neri C, Bellacchio E (2005). NF1 gene mutations represent the major molecular event underlying neurofibromatosis-Noonan syndrome. Am J Hum Genet.

[17] Legius E, Schrander-Stumpel C, Schollen E, Pulles-Heintzberger C, Gewillig M, Fryns JP (2002). PTPN11 mutations in LEOPARD syndrome. J Med Genet.

[18] Razzaque MA, Nishizawa T, Komoike Y, Yagi H, Furutani M, Amo R (2007). Germline gain-of-function mutations in RAF1 cause Noonan syndrome. Nat Genet.

[19] Zhang J, Tong H, Fu X, Zhang Y, Liu J, Cheng R (2015). Molecular characterization of NF1 and Neurofibromatosis type 1 genotype-phenotype correlations in a Chinese population. Sci Rep.

